# Postoperative delirium in 47 379 individuals undergoing transcatheter aortic valve replacement: a systematic review and meta-analysis

**DOI:** 10.1097/MS9.0000000000001096

**Published:** 2023-07-26

**Authors:** Sidhant Ochani, Alishba Adnan, Amna Siddiqui, Asifa Kalwar, Sandhaya Kukreja, Mushtaq Ahmad, Muhammad Hasan Ashraf, Mustafa Ali Asghar

**Affiliations:** a Department of Medicine, Khairpur Medical College, Khairpur Mir’s; b Department of Medicine, Karachi Medical and Dental College; c Department of Medicine, Dow University of Health Sciences; d Department of Medicine, Ziauddin Medical University, Karachi, Pakistan

**Keywords:** CAM, confusion assessment method, delirium, stroke, TAVR, transcatheter aortic valve replacement

## Abstract

**Objective:**

The study aims to discuss the assessment methods used for the incidence of in-hospital postoperative delirium (IHPOD) in transcatheter aortic valve replacement (TAVR) patients and explore possible strategies for preventing and reducing postoperative complications in the geriatric population.

**Methodology:**

An electronic search of PubMed, Embase, BioMedCentral, Google Scholar, and the Cochrane Central Register of Controlled Trials was conducted up to August 2021, to identify studies on the IHPOD following TAVR in patients above 70 years. The primary objective of the study was to determine the incidence of delirium following TAVR and procedures like transfemoral (TF) and non-TF approaches. The secondary objectives were to determine the incidence of stroke and incidence according to the confusion assessment method (CAM) diagnostic tool. The authors only included studies published in English and excluded patients with comorbidities and studies with inaccessible full-text.

**Results:**

Among the selected 42 studies with 47 379 patients, the incidence of IHPOD following TAVR was 10.5% (95% CI: 9.2–11.9%, *I*^2^=95.82%, *P*<0.001). Incidence based on CAM was 15.6% (95% CI: 10.5–20.7%, *I*^2^=95.36%, *P*<0.001). The incidence of IHPOD after TF-TAVR was 9.3% (95% CI: 7.6–11.0%, *I*^2^=94.52%, *P*<0.001), and after non-TF TAVI was 25.3% (95% CI: 15.4–35.1%, *I*^2^=92.45%, *P*<0.001). The incidence of stroke was 3.7% (95% CI: 2.9–4.5%, *I*^2^=89.76%, *P*<0.001). Meta-regression analyses between mean age (*P*=0.146), logistic EuroSCORE (*P*=0.099), or percentage of participants treated using the TF approach (*P*=0.276) were nonsignificant while stroke (*P*=0.010) was significant. When considering these variables, the residual heterogeneity remained high indicating that other variables influence the heterogeneity.

**Conclusion:**

IHPOD following TAVR was observed in 10.5% of individuals and in 15.6% using CAM. Its incidence was found to be three times higher after non-TF TAVR (25.3%) compared to TF TAVR (9.3%). Stroke showed an incidence of 3.7% after TAVR and was found to be significantly associated with the risk of developing delirium following TAVR. Further studies are needed to evaluate possible causes and risk factors responsible for delirium and to assess the role of anesthesia and cerebral embolic protection in preventing delirium after TAVR.

## Introduction

Highlights

- We evaluated the incidence of in-hospital postoperative delirium after transcatheter aortic valve replacement (TAVR).
- In-hospital postoperative delirium following TAVR was observed in 10.5% of individuals and in 15.6% using confusion assessment method.
- Its incidence was found to be 2.72 times higher after nontransfemoral TAVR (25.3%) compared to transfemoral TAVR (9.3%).
- Further studies are needed to evaluate possible causes and risk factors responsible for delirium and to assess the role of anesthesia and cerebral embolic protection in preventing delirium after TAVR.

Aortic stenosis (AS) is a hemodynamically significant narrowing of the left ventricle’s outflow and is categorized as valvular, subvalvular, or supra-valvular depending on the degree of obstruction^1^. It has an incidence of 2% in the population aged 65 years or older^2^. Being a common valvular disorder, it can lead to left ventricular outflow obstruction^3^. Common causes include rheumatic disease, congenitally abnormal, and calcified valves. It becomes symptomatic after a decade and presents as exertional dyspnea or fatigue^4^. The natural progression of the disease cannot be prevented but aortic valve replacement (AVR) provides symptomatic relief and improves survival. Unoperated and symptomatic patients have a mortality rate of up to 50% over 2 years^5^. AVR is recommended in mild symptomatic, asymptomatic severe, and severe symptomatic AS. AVR can be done surgically or via a transcatheter approach^6^. Elderly patients are at increased risk of postoperative complications or death after surgical AVR. These patients require less invasive treatment, such as transcatheter AVR. AVR through blood vessels is known as transcatheter aortic valve replacement (TAVR). Transfemoral (TF) (in the upper leg) or nontransfemoral (non-TF) routes like transapical (through the heart wall), subclavian (below the collar bone), direct aortic (through a minimally invasive surgical incision into the aorta), and transcaval (from a temporary hole in the aorta near the umbilicus through a vein in the upper leg) are some of the access methods used to deliver the replacement valve^7^. The benefits of TAVR are that it is a less invasive surgery, has a quicker recovery, a shorter hospital stay, higher one-year survival rates, a lower stroke rate, a reduced risk of rehospitalization within a year^8^ and lower rates of postoperative delirium as compared to surgical aortic valve replacement^9^. Despite its success, it has a variety of complications that may increase morbidity, necessitate immediate surgical intervention, or even result in death. These include cerebrovascular events, vascular complications, myocardial infarction, valve regurgitation and malpositioning, and many more. The risk of these complications significantly decreased as a result of advancements in procedural techniques, medical equipment’s growing experience, and improvements in patients’ imaging^10^. Neurological abnormalities ranging from mild cognitive abnormalities to postoperative delirium and, rarely, stroke if they occur, are one of the most dreadful complications after TAVR^11^. Postoperative delirium can be described as an acute and fluctuating neurologic disorder that reflects an alteration from baseline cognition and is characterized by the important features of inattention and disorganized thinking^12^. It is usually diagnosed through CAM-ICU (Confusion Assessment Method for the Intensive Care Unit)^13^. It is associated with increased mortality and prolonged hospital stay^14^. More than a year following surgery, postoperative delirium can signal the onset of long-term and likely permanent cognitive impairment^15^. Individual characteristics, care settings, and the sensitivity of the detection method all influence the incidence and prevalence of delirium. Delirium affects 1–2% of the community-dwelling population, but it can reach up to 14% in patients aged 85 years and older and 14–24% in those admitted to the hospital^16^. Its incidence is increased in hospitalized patients, more than 50% in the ICU, 60% in nursing homes and postacute care, and 83% near the end of life^17^. According to the literature, the incidence of in-hospital postoperative delirium (IHPOD) following TAVR ranges from 0 to 44%, with non-TF TAVR having the greatest incidence rate. There is evidence that delirium in older hospitalized patients can be avoided in 20–30% of cases^18^. Inouye *et al*.^19^ in JAMA raised concern about ageism and the increasing geriatric population to threefold by 2050. Hence, we conducted this meta-analysis to improve disease outcomes for the geriatric population. The purpose of this study is to determine the incidence of IHOPD and stroke, which is underreported after TAVR, to discuss assessment methods used for detecting IHPOD in TAVR patients, and explore possible strategies for preventing and reducing postoperative complications in the largest geriatric population. A differential diagnosis of delirium is described in Table 1.

**Table 1:** Differential diagnosis of delirium.

Symptoms	Delirium	Dementia	Depression	Psychosis
Descriptive features	Confusion and inattention	Memory loss	Sadness and anhedonia	Loss of contact with reality
Onset	Rapid, hours to days	Progressive, develops over several years	Rapid or slow	Rapid, days to weeks
Course	Fluctuating usually worse at night	Progressive	Chronic, usually worse in the morning	Chronic
Reversibility	Usually reversible	Irreversible	Usually reversible	Usually reversible
Attention and concentration	Reduced	Generally intact, but may be impaired	May be reduced	Generally intact, but may be impaired
Level of consciousness and orientation	Fluctuates, disoriented	Impaired, worsening in the late stages	Generally normal	Generally normal, individual may be disoriented in acute stage
Memory	Poor short-term memory	Loss of short-term memory	Memory intact	Memory intact
Cognition	Focal cognitive failure	Global cognitive failure	Cognition intact	Variable
EEG	Generalized diffuse slowing	Variable	Generally normal	Generally normal

## Methods

### Search strategy and data sources

This meta-analysis was performed according to the preferred reporting items for systematic reviews and meta-analyses (PRISMA) guidelines^20^. An electronic search of PubMed, Embase, BioMedCentral, Google Scholar, and the Cochrane Central Register of Controlled Trials was conducted up to August 2021, using the search strategy created using the Boolean operators: *“TAVR” OR “TAVI” OR “transcatheter aortic valve implantation” OR “transcatheter aortic valve” OR “aortic valve replacement” OR “transcatheter aortic valve replacement” AND “delirium” OR “cognition disorders” OR “cognition” OR “acute confusional state” OR “acute brain failure” OR “acute brain dysfunction” OR “encephalopathy”.* A comprehensive search of the English-language medical literature was done by two authors (A.A. and A.S.) independently using the databases mentioned previously. In case of any conflicts or doubts were discussed and resolved with a third author (S.O.).

### Study selection

We registered PROSPERO^21^ (CRD42021281896) in which we mentioned that the following eligibility criteria were used to select studies: (a) octogenarian patients or 70 above; (b) no review articles; (c) may have AS and have undergone TAVI; the (d) main outcome of delirium must be a significant incidence in that patient cohort; (e) we will look at particular measures, for example, CAM, Richmond Agitation-Sedation Scale (RASS), Mini-Mental State Examination (MMSE), etc. to determine delirium among all studies; (f) exclude multiple surgery patients; (g) exclude patients with other neurological illnesses that impair cognition, for example, Alzheimer’s; (h) articles showed to be in English; (i) should have access to full-text articles.

### Data extraction

Articles were assessed by two independent authors (A.A. and A.S.). Duplicate articles were removed by using Zotero. Articles were shortlisted based on title, abstract, and then full-text. Any conflicts and confusion regarding the data extraction were discussed and resolved with a third author (S.O.). In the case of overlapping populations, the most relevant article or the one with the highest sample size was chosen. From these selected studies relevant data on authors, year of publication, sample size, study design, observed event rates, Euro-scores, diagnostic methods, and baseline participant clinical characteristics were extracted and are given in Table 2.

**Table 2:** Study participant characteristics.

References	Year	Design	Patients (*n*)	Age	Male (%)	LES%	Transfemoral access (*n*)	Diagnostic Method	Frequency Examined (*n*)	Time Period	Incidence of POD (%)
Walther^22^	2010	Propensity matched	100	82.7±5.0	23	29.4±13.0	0	NA	NA	NA	3.0
Abdel- Wahab^23^	2011	Prospective	690	81.4±6.3	44	20.4±13.1	638	NA	NA	NA	9.3
Erdoes^24^	2011	Prospective	44	78.6±6.0	55	28.0±15.0	32	CAM	NA	Preoperative, Postprocedural days 1 and 4–6	0
Wilbring^25^	2013	Propensity matched	53	77.8±4.5	65	29.9±14.0	0	NA	NA	NA	11.5
Sherif^26^	2014	Prospective	1432	Female: 82.8±5.8; Male: 80.3±6.4	42	NA	1256	NA	NA	NA	9.2
Tse^27^	2014	Retrospective	117	81.0±8.0	50	N/A	74	Physician diagnosis; DSM 4	NA	NA	27
Santarpino^28^	2015	Propensity matched	102	80.0±4.0	41	17.0±14.0	NA	NA	NA	NA	2
Bestehorn^29^	2015	Propensity matched	763	78.8±6.0	56	13.5	763	Physician diagnosis	NA	NA	3.8
Eide^30^	2015	Prospective	65	84.8±2.8	37	19.6	NA	CAM	Daily	5 days	44.6
Egerod^31^	2015	Prospective	54	79.0±7.3	52	N/A	52	CAM-ICU	2	NA	0
Gauthier^32^	2015	Retrospective	176	84.0–86.0	52	NA	117	NA	NA	NA	26.1
Jagielak^33^	2015	Prospective	32	80.9±5.2	47	2.8	0	NA	NA	NA	6.5
Adrie^34^	2015	Propensity matched	26	86.0 (83–89)	57	32.0	26	CAM-ICU	NA	NA	0
Van Mieghem^35^	2016	Retrospective	65	82.0 (78–85)	52	NA	65	MMSE & MoCA	NA	1 day preoperative, 5–7 days postoperative	9.0
Nijenhuis^36^	2016	Prospective	591	80.2±8.4	42	NA	337	NA	NA	NA	7.1
Chu^37^	2016	Prospective	30	85.0±5.5	37	NA	0	NA	NA	3	3.3
Eggebrecht^38^	2016	Prospective	17,919	81.2±6.1	45	21.1±15.4	17919	NA	NA	NA	3.8
Huded^39^	2016	Retrospective	294	84.3±6.5	53	N/A	205	CAM, clinician diagnosis	Twice daily	NA	20.7
Maniar^40^	2016	Retrospective	168	81.0±8.0	45	N/A	57	CAM-ICU	Every 12 hours	NA	29.2
Abawi^41^	2016	Retrospective	268	80.0±7.0	46	18.0±9.0	228	DOS	At the end of every shift	Up till discharge	13.4
Fanning^42^	2016	Prospective	40	81.7±6.9	40	N/A	20	CAM, MoCa	3±1 days postoperative	6 months	2.5
Schoenenberger^43^	2016	Prospective	229	83.4±5.5	44	17.6±15.8	213	Physician diagnosis	NA	3 months preoperative, 6 months postoperative	0.9
Serletis-Bizios^44^	2016	Prospective	130	84.7±5.4	52	15.3±8.5	130	NA	NA	NA	4.6
Assmann^45^	2016	Retrospective	89	80.4±6.3	43	15.9±9.8	89	DOS	Daily	During hospital stay	28.0
Soundhar^46^	2017	Retrospective	7566	81.0	51	NA	NA	ICD-9-CM	NA	NA	4.6
Frerker^47^	2017	Propensity matched	805	77.5±4.4	40	NA	805	NA	NA	NA	2.5
Boureau^48^	2017	Prospective	150	83.7±4.6	56	17.0±8.1	NA	NA	NA	NA	12.0
van Mourik^49^	2017	Retrospective	114	79.6±8.7	32	17.6±11.4	114	NA	NA	NA	4.4
Giuseffi^9^	2017	Retrospective	105	79.9±9.5	52	N/A	105	CAM-ICU	Every 12 h	3 days postoperative or up till discharge	19.0
Bagienski^50^	2017	Retrospective	141	82.0 (77.5– 85.0)	37	14.0	113	Chart based	NA	First 4 days after index procedure	20.6
Fanning^51^	2017	Prospective	31	82.4±7.7	35	16.7±13.2	31	CAM	Daily	During hospital stay	3.2
Stachon^52^	2018	Nationwide cohort	11560	82.2±5.7 (TF-AVR) 81.3±5.3 (TAVR)	TF-TAVR 53 TAVR 67	TF-TAVR (23±15) TAVR (25±15)	9038	ICD-10-GM	NA	NA	8
Abawi^53^	2018	Observational	30	81.0±6.0	50	19.0±10	47	DOS	Daily at the end of every shift	During hospital stay and 4 months postoperative	17.0
Shi^54^	2019	Prospective	187	83.7±6.0	48	NA	NA	CAM	Daily during day shift between (12pm–6pm)	From postoperative day 1 till the day before discharge	25.5
Wulp^55^	2019	Preoperative	511	80.0 (76-84)	45	13.1 (8.9–21.3)	108	DSM 4	3 times a day	From postoperative day 1 up till discharge	14.10
Wulp^56^	2019	Prospective	703	80.0 (75–84)	48	13.8 (8.9–21.6)	175	CAM	3 times a day on daily basis	NA	16.50
Beishuizen^57^	2020	Prospective	91	83.6±4.0	50	19.0 (12.9–22.3)	75	DSM-5	Daily during weekdays	During hospital stay & 6–12 months postoperative	15.40
Goudzwaard^58^	2020	Prospective	543	79.1 ± 8.0	55	N/A	492	CGA, psychiatric examination	Once a day on daily basis	From admission up till 4 days postoperative	14
Rao^59^	2020	Prospective	110	81.3±6.4	52	N/A	N/A	CAM-ICU	Once a day on daily basis	From postoperative day 1 till the day before discharge	25.5
Mauri^60^	2021	Prospective	661	82±6.6	49	N/A	644	CAM-ICU	1st and 2nd postoperative day for every patient and up to 7 days in case of suspected delirium	Within 1 year	10.0
Humbert^61^	2021	Prospective	93	82.1±10.3	55	NA	NA	CAM	Postoperative days 1, 2, 3, and 7.	3 month follow-up	23.0
Luque^62^	2021	Cohort	501	82.9±5.8	42	N/A	501	CAM	Every 8 h daily	Follow-up 24 months postoperative	22.0

### Outcomes

The main objective of our meta-analysis was to discover the incidence of IHPOD following TAVR. IHPOD was explained as the presence of delirium during a hospital stay following the AS treatment. Secondary results were the incidence of IHPOD stated by using a specific diagnostic tool (e.g. CAM) and the incidence of IHPOD according to procedure (TF TAVR vs. non-TF TAVR). The impact of baseline characteristics [age, TF approach, Logistic Euro SCORE (LES)] on the incidence of IHPOD following TAVR was also investigated. We were unable to examine the effect of general anesthesia against local anesthesia or of a cerebral protective device on the incidence of IHPOD following TAVR due to a lack of data.

### Quality assessment

Two independent authors (H.A. and M.A.) assessed the risk of bias for the included studies using the NIH Quality Assessment Tool for Observational Cohort and Cross-Sectional Studies^63^. Its criteria had 14 questions that were applied to every individual study answering yes, no, not applicable, not reported, and cannot determine, followed by a quality rating of low, fair, and good (Table 3). Any conflicts and confusion regarding the data extraction were discussed and resolved with a third author (S.O.). Furthermore, the quality of evidence was graded as very low, low, moderate, or high using the Grades of Recommendation, Assessment, Development, and Evaluation (GRADE) assessment tool based on the risk of bias, publication bias, imprecision, inconsistency, and indirectness (Table 4) ^64^. A self-evaluation of the quality of the systematic review and meta-analysis was done using the AMSTAR 2 criteria^65^.

**Table 3:** Risk of bias using the NIH quality assessment tool for observational cohort and cross-sectional studies

References	1. Was the research question or objective in this paper clearly stated?	2. Was the study population clearly specified and defined?	3. Was the participation rate of eligible persons at least 50%?	4. Were all the subjects selected or recruited from the same or similar populations (including the same time period)? Were inclusion and exclusion criteria for being in the study pre-specified and applied uniformly to all participants?	5. Was a sample size justification, power description, or variance and effect estimates provided?	6. For the analyses in this paper, were the exposure(s) of interest measured prior to the outcome(s) being measured?	7. Was the time frame sufficient so that one could reasonably expect to see an association between exposure and outcome if it existed?	8. For exposures that can vary in amount or level, did the study examine different levels of the exposure as related to the outcome (e.g. categories of exposure, or exposure measured as continuous variable)?	9. Were the exposure measures (independent variables) clearly defined, valid, reliable, and implemented consistently across all study participants?	10. Was the exposure(s) assessed more than once over time?	11. Were the outcome measures (dependent variables) clearly defined, valid, reliable, and implemented consistently across all study participants?	12. Were the outcome assessors blinded to the exposure status of participants?	13. Was loss to follow-up after baseline 20% or less?	14. Were key potential confounding variables measured and adjusted statistically for their impact on the relationship between exposure(s) and outcome(s)?	Overall Quality
Walther^22^	Yes	Yes	Yes	Yes	No	Yes	CD	NA	Yes	No	Yes	NA	Yes	Yes	Good
Abdel-Wahab^23^	Yes	Yes	Yes	Yes	No	Yes	CD	NA	Yes	No	Yes	NA	Yes	Yes	Good
Erdoes^24^	Yes	Yes	CD	Yes	CD	Yes	Yes	NA	Yes	Yes	NA	NA	Yes	Yes	Fair
Wilbring^25^	Yes	Yes	Yes	Yes	No	Yes	CD	NA	Yes	No	Yes	NA	Yes	Yes	Good
Sherif^26^	Yes	Yes	CD	Yes	CD	Yes	Yes	NA	Yes	Yes	NA	NA	Yes	Yes	Good
Tse^27^	Yes	Yes	Yes	Yes	No	Yes	CD	NA	Yes	No	Yes	NA	Yes	Yes	Good
Santarpino^28^	Yes	Yes	Yes	Yes	No	Yes	CD	NA	Yes	No	Yes	NA	Yes	Yes	Good
Bestehorn^29^	Yes	Yes	Yes	Yes	No	Yes	CD	NA	Yes	No	Yes	NA	Yes	Yes	Good
Eide^30^	Yes	Yes	Yes	Yes	No	Yes	CD	NA	Yes	No	Yes	NA	Yes	Yes	Good
Egerod^31^	Yes	Yes	Yes	Yes	No	Yes	CD	NA	Yes	No	Yes	NA	Yes	Yes	Good
Gauthier^32^	Yes	Yes	Yes	Yes	No	Yes	CD	NA	Yes	No	Yes	NA	Yes	Yes	Good
Jagielak^33^	Yes	Yes	CD	Yes	CD	Yes	Yes	NA	Yes	Yes	NA	NA	Yes	NR	Fair
Adrie^34^	Yes	Yes	Yes	Yes	No	Yes	CD	NA	Yes	No	Yes	NA	Yes	Yes	Good
Van Mieghem^35^	Yes	Yes	Yes	Yes	No	Yes	CD	NA	Yes	No	Yes	NA	Yes	Yes	Good
Nijenhuis^36^	Yes	Yes	Yes	Yes	No	Yes	Yes	NA	Yes	No	Yes	NA	Yes	Yes	Low
Chu^37^	Yes	Yes	Yes	Yes	No	Yes	Yes	NA	No	Yes	No	NA	Yes	Yes	Low
Eggebrecht^38^	Yes	Yes	Yes	Yes	No	Yes	Yes	NA	Yes	No	Yes	NA	Yes	Yes	Low
Huded^39^	Yes	Yes	Yes	Yes	No	Yes	CD	NA	Yes	No	Yes	NA	Yes	Yes	Fair
Maniar^40^	Yes	Yes	Yes	Yes	No	Yes	CD	NA	Yes	No	Yes	NA	CD	Yes	Fair
Abawi^41^	Yes	Yes	Yes	Yes	No	Yes	Yes	NA	Yes	No	Yes	NA	CD	Yes	Fair
Fanning^42^	Yes	Yes	Yes	Yes	No	Yes	CD	NA	Yes	No	Yes	NA	No	Yes	Low
Schoenenberger^43^	Yes	Yes	Yes	Yes	No	Yes	Yes	NA	Yes	No	Yes	NA	Yes	Yes	Low
Serletis-Bizios^44^	Yes	Yes	Yes	Yes	No	Yes	CD	NA	Yes	No	Yes	NA	Yes	Yes	Low
Assmann^45^	Yes	Yes	Yes	Yes	No	Yes	CD	NA	Yes	No	Yes	NA	Yes	CD	Fair
Soundhar^46^	Yes	Yes	Yes	Yes	No	Yes	Yes	NA	Yes	No	Yes	NA	Yes	Yes	Low
Frerker^47^	Yes	Yes	Yes	Yes	No	Yes	CD	NA	Yes	No	Yes	NA	NR	Yes	Low
Boureau^48^	Yes	Yes	Yes	Yes	No	Yes	CD	NA	Yes	No	Yes	NA	No	Yes	Low
Mourik^49^	Yes	Yes	Yes	Yes	No	Yes	Yes	NA	Yes	No	Yes	NA	Yes	Yes	Low
Giuseffi^9^	Yes	Yes	Yes	Yes	No	Yes	Yes	NA	Yes	No	Yes	NA	CD	Yes	Fair
Bagienski^50^	Yes	Yes	Yes	Yes	No	Yes	Yes	NA	Yes	No	Yes	NA	Yes	Yes	Low
Fanning^51^	Yes	Yes	Yes	Yes	No	Yes	Yes	NA	Yes	No	Yes	NA	Yes	Yes	Low
Stachon^52^	Yes	No	Yes	CD	No	Yes	CD	NA	Yes	CD	Yes	NA	CD	Yes	Fair
Abawi^53^	Yes	Yes	Yes	Yes	No	Yes	Yes	NA	Yes	No	Yes	NA	No	Yes	Fair
Shi^54^	Yes	Yes	Yes	Yes	No	Yes	Yes	NA	Yes	No	Yes	NA	Yes	Yes	Low
Wulp^55^	Yes	Yes	Yes	Yes	No	Yes	Yes	NA	Yes	No	Yes	NA	Yes	Yes	Low
Wulp^56^	Yes	Yes	Yes	Yes	No	Yes	Yes	NA	Yes	No	Yes	NA	Yes	Yes	Low
Beishuizen^57^	Yes	Yes	Yes	Yes	No	Yes	Yes	NA	Yes	No	Yes	NA	Yes	Yes	Low
Goudzwaard^58^	Yes	Yes	Yes	Yes	No	Yes	Yes	NA	Yes	No	Yes	NA	CD	Yes	Fair
Rao^59^	Yes	Yes	Yes	Yes	No	Yes	Yes	NA	Yes	No	Yes	NA	Yes	Yes	Low
Mauri^60^	Yes	Yes	Yes	Yes	No	Yes	Yes	NA	Yes	No	Yes	NA	CD	Yes	Fair
Humbert^61^	Yes	Yes	No	CD	No	Yes	CD	NA	Yes	CD	Yes	NA	No	Yes	Fair
Luque^62^	Yes	Yes	Yes	Yes	No	Yes	CD	NA	Yes	No	Yes	NA	No	Yes	Fair

**Table 4:** Grading of recommendations assessment, development, and evaluation (GRADE) summary of findings.

Outcome	Number of participants (studies)	Effect estimate (95% CI)	Risk of bias	Inconsistency	Indirectness	Imprecision	Publication bias	Quality of evidence (GRADE)
TAVR	47 379	0.105 (0.092– 0.119)	Serious	Serious	Not serious	Serious	NA	Low ⊕⊝⊝⊝
TF-TAVR	31 536	0.093 (0.076– 0.110)	Not serious	Serious	Not serious	Serious	NA	Moderate ⊕⊕⊝⊝
Non-TF TAVR	580	0.253 (0.154– 0.351)	Not serious	Serious	Not serious	Serious	NA	Moderate ⊕⊕⊝⊝
CAM	3082	0.156 (0.105– 0.207)	Not serious	Serious	Not serious	Serious	NA	Moderate ⊕⊕⊝⊝
Stroke	1141	0.071 (0.052– 0.091)	Serious	Serious	Not serious	Serious	NA	Low ⊕⊝⊝⊝

CAM, confusion assessment method; TF, transfemoral; TAVR, transcatheter aortic valve replacement.

### Statistical analysis

A meta-analysis involving only single-arm studies that stated the IHPOD following TAVR was conducted. Cumulative event rates from these studies were extracted. A binary random effects model was utilized to calculate the pooled estimates and 95% CI^66^. The *I*^2^ statistic was used to assess heterogeneity across studies, with a value of *I*^2^ between 25 and 50% considered mild heterogeneity, between 50 and 75% considered moderate heterogeneity, and greater than 75% considered severe heterogeneity^67^. Studies stating the incidence of IHPOD using CAM were assessed via a subgroup analysis. Another subgroup analysis was conducted to evaluate the incidence of IHPOD in participants undergoing different vascular accesses (TF, non-TF). Studies reporting the rate of IHPOD according to TF or non-TF approaches were included in this subgroup analysis. A meta-regression analysis involving a random effect model was performed to assess the impact of Logistic EuroScore, age, and the percentage of participants treated using the TF approach on the incidence of IHPOD following TAVR. These factors were also taken into consideration when evaluating heterogeneity. Statistical analyses were performed using the latest version of OpenMeta [Analyst].

## Results

### Literature search results

**Figure 1:**
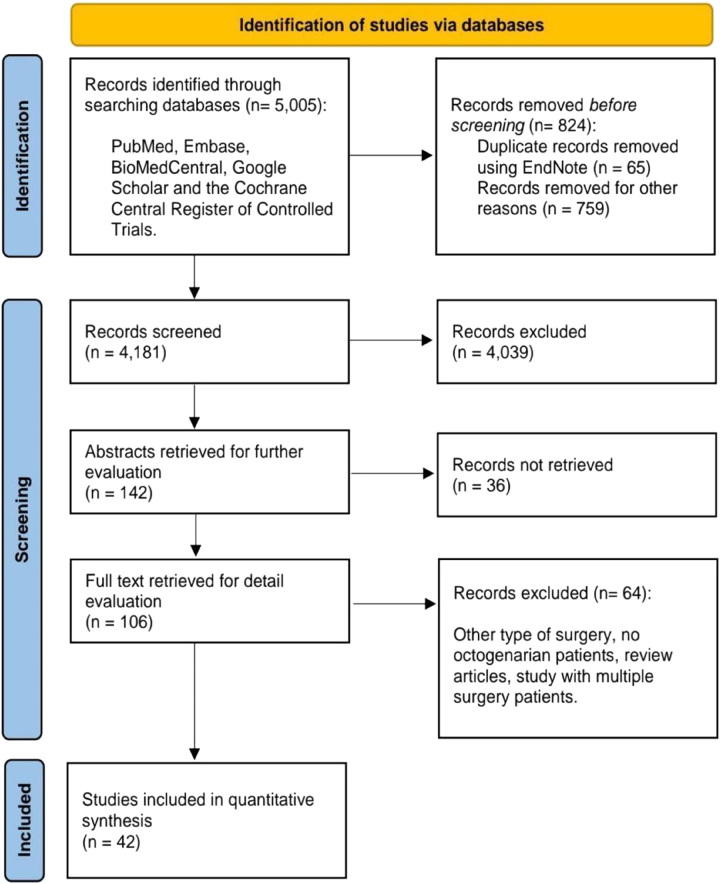
PRISMA (Preferred Reporting Items for Systematic reviews and Meta-Analyses) flowchart.

Initial searches of the aforementioned five electronic databases yielded 5005 potential studies, from which 824 duplicates were removed. The remaining 4181 results were screened, of which 4039 articles failed to meet the inclusion criteria. For further evaluation, abstracts for the remaining 142 articles were then reviewed, of which 36 records were not accessible. Later, based on a full-text review of the remainder of the 106 results, 64 results were excluded from the study due to ineligibility. After exclusions, a total of 42 studies remained for analysis (as shown in Table 2)^9,22–62^. The PRISMA flowchart Figure 1 summarizes the results of our literature search.

### Quality assessment of included studies

Assessment of the risk of bias using the NIH Quality Assessment Tool for Observational Cohort and Cross-Sectional Studies found good quality in 12 studies, fair quality in 13 studies, and low quality in 17 studies. The most common issue was the sample size and with no justification provided followed by a lack of follow-up and exposure assessment more than once or over time.

### Results of meta-analysis

#### Delirium

**Figure 2:**
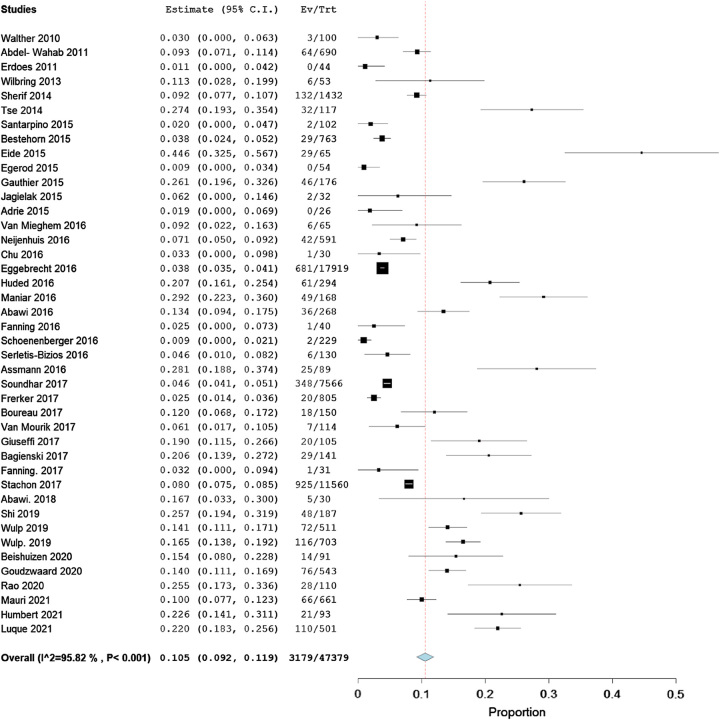
Forest plot showing individual and pooled event rates for in-hospital postoperative delirium after transcatheter aortic valve replacement from included studies.

Among the selected 42 studies, in total having 47 379 patients, the incidence of IHPOD following TAVR varied from 0 to 44.6% with a pooled estimate rate of 10.5% (95% CI: 9.2–11.9%). The heterogeneity across the studies was high (*I*^2^=95.82%, *P*<0.001; Fig. 2). The quality of the evidence was judged to be low, with serious concerns about the risk of bias in the included studies (Table 4).

#### Confusion assessment method

**Figure 3:**
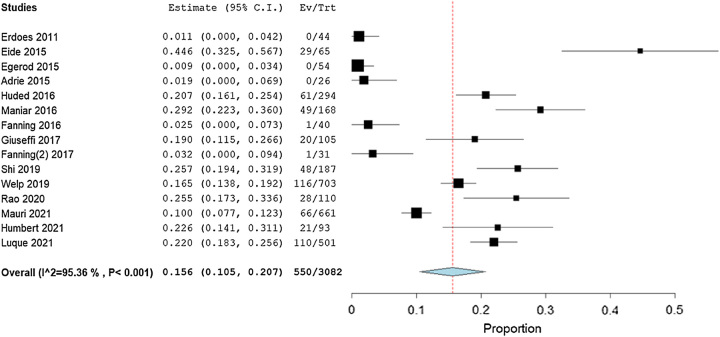
Forest plot showing incidence of in-hospital postoperative delirium after transcatheter aortic valve replacement defined using Confusion Assessment Method.

Fifteen studies, in total having 3082 patients, stated the IHPOD according to the CAM. The pooled estimate rate of these studies was 15.6% (95% CI: 10.5–20.7%) with high heterogeneity (*I*^2^=95.36%, *P*<0.001; Fig. 3). The quality of the evidence was judged to be moderate, with no serious concerns about the risk of bias in the included studies (Table 4).

#### Transfemoral transcatheter aortic valve replacement

**Figure 4:**
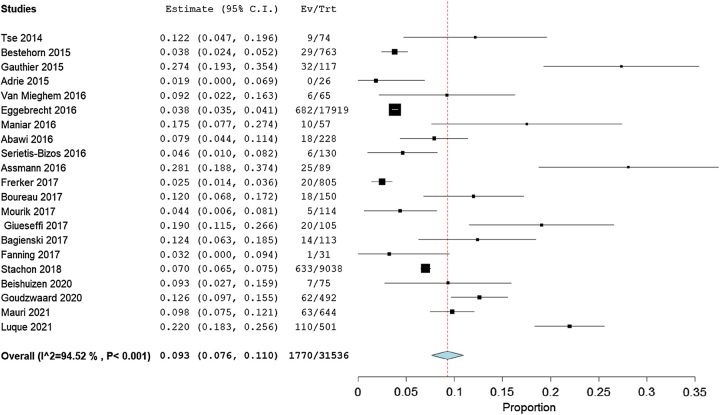
Incidence of in-hospital postoperative delirium after TF-TAVR (transfemoral-transcatheter aortic valve replacement).

Twenty-one studies, in total having 31 536 patients, stated the incidence of IHPOD after TF-TAVR. The pooled estimate rate of these studies was 9.3% (95% CI: 7.6–11.0%) with evidence of high heterogeneity (*I*^2^=94.52%, *P*<0.001; Fig. 4). The quality of the evidence was judged to be moderate, with no serious concerns about the risk of bias in the included studies (Table 4).

#### Nontransfemoral transcatheter aortic valve replacement

**Figure 5:**
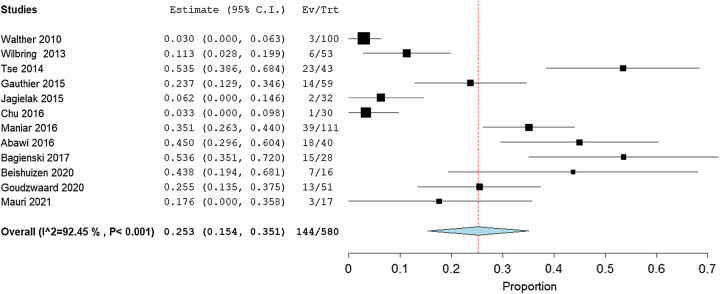
Forest plot showing incidence of in-hospital postoperative delirium after non-TF TAVR (transfemoral transcatheter aortic valve replacement).

Twelve studies, in total having 580 patients, stated the incidence of IHPOD after non-TF TAVR. The pooled estimate rate of these studies was 25.3% (95% CI: 15.4–35.1%) with the lower bound of the 95% CI higher than the upper bound of the 95% CI estimated for IHPOD after non-TF TAVR. Heterogeneity among studies was high for non-TF TAVR (*I*^2^=92.45%, *P*<0.001; Fig. 5). The quality of the evidence was judged to be moderate, with no serious concerns about the risk of bias in the included studies (Table 4).

#### Stroke

**Figure 6:**
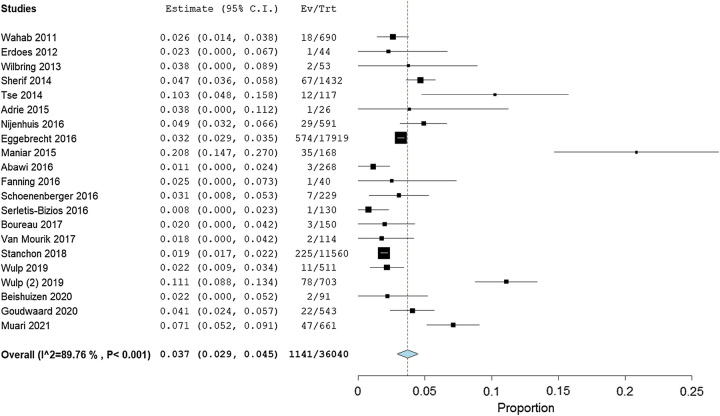
Forest plot showing incidence of Stroke after transcatheter aortic valve replacement.

Twenty-one studies, in total having 1141 patients, stated the incidence of stroke after TAVR. The pooled estimate rate of these studies was 3.7% (95% CI: 2.9–4.5%) with evidence of high heterogeneity (*I*^2^=89.76%, *P*<0.001; Fig. 6). The quality of the evidence was judged to be low, with serious concerns about the risk of bias in the included studies (Table 4).

**Figure 7:**
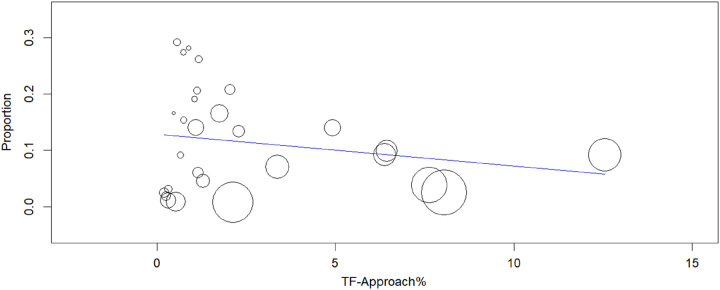
Meta-regression analysis: effect of TF (transfemoral) approach on in-hospital postoperative delirium after TAVR (transcatheter aortic valve replacement) (*P*=0.276).

**Figure 8:**
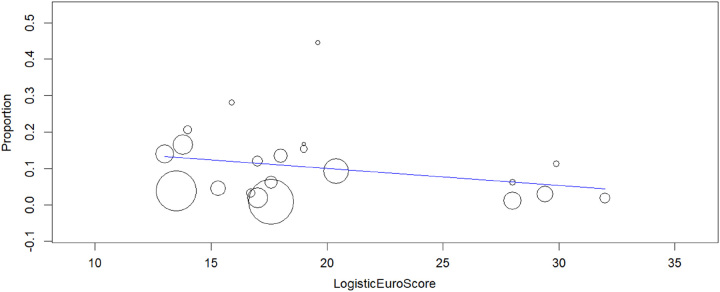
Meta-regression analysis: effect of Logistic EuroSCORE on in-hospital postoperative delirium after TAVR (transcatheter aortic valve replacement) (*P*=0.133).

**Figure 9:**
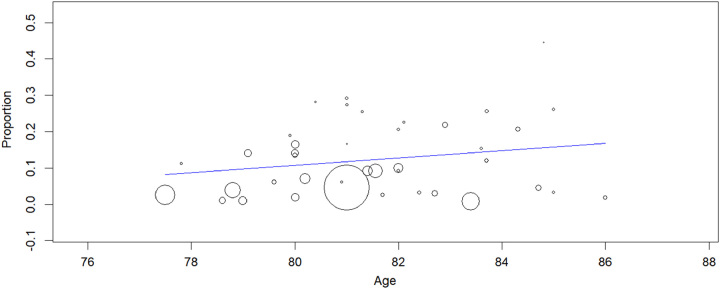
Meta-regression analysis: effect of age on in-hospital postoperative delirium after TAVR (transcatheter aortic valve replacement) (*P*=0.150).

**Figure 10:**
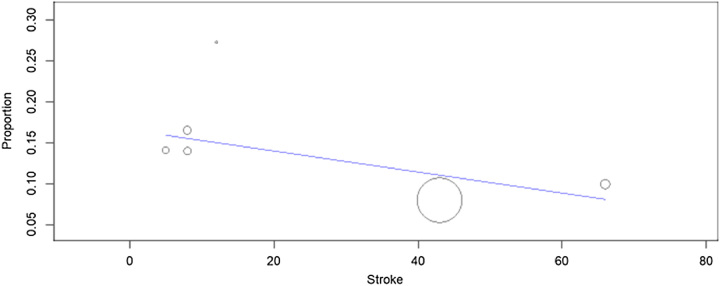
Meta-regression analysis: effect of stroke on in-hospital postoperative delirium after TAVR (transcatheter aortic valve replacement) (*P*=0.010).

The relationship observed in the meta-regression analyses (Figs 7–10) between mean age (*P*=0.150), logistic EuroSCORE (*P*=0.133), or percentage of participants treated using the TF approach (*P*=0.276) were nonsignificant while the effect of stroke (*P*=0.010) was found significant, which showed stroke was associated with the risk of developing delirium following TAVR. When considering these variables, the residual heterogeneity remained high indicating that other variables influence the heterogeneity.

## Discussion

The main results of our meta-analysis of 42 studies comprising 47 379 participants report the pooled incidence of IHPOD after TAVR to be 10.5% (95% CI: 9.2–11.9%). This study verifies that IHPOD is frequently observed after TAVR (0–44.6%). The diagnostic accuracy and sensitivity were further increased by using CAM with an even higher incidence of IHPOD of 15.6% (95% CI: 10.5–20.7%). This test is for healthcare workers who are not equipped with the advanced knowledge of psychiatry, to detect delirium quickly.

When finding the incidence of stroke, it was found that stroke was associated with the risk of developing delirium as a contributing factor, and was seen in about 3.7% of patients undergoing TAVR.

To compare the pooled incidence of the two procedures, IHPOD after non-TF TAVR [25.3% (95% CI: 15.4–35.1%)] was 2.72 times as high as with TF-TAVR [9.3% (95% CI: 7.6–11.0%)]. No significant relationships were observed on performing meta-regression analyses between; the incidence of IHPOD after TAVR, mean age, logistic EuroSCORE, or percentage of participants treated using TF access. We found that the incidence of IHPOD in individuals undergoing TAVR ranged between 0 and 44.6%. Intriguingly, while the larger study in our meta-analysis reported a lower incidence of IHPOD after TAVR like Eggebrecht^38^ (3.8%), Soundhar^46^ (4.6%), and Stachon^52^ (8%), and on the other hand small studies like Eide^30^ (44.6%), Assman^45^ (28.1%), and Tse^27^ (27.4%) claimed a higher incidence of IHPOD after TAVR. The reasons behind the difference in incidence reported by these studies are as follows: They could depend on participant factors including age at admission, comorbid condition, and TAVR access. While the hospital factors like the efficacy of data collection, hospital complication rates, and follow-up rates.

Predisposing factors, such as age, pre-existing cognitive impairment, and cerebrovascular disease, are not modifiable and reflect a person’s vulnerability to delirium. In contrast, precipitating factors, including major surgery, psychoactive drugs, transcatheter valvular interventions, infectious disease, and metabolic alterations, are potentially modifiable factors that trigger the onset of delirium^41,67,68^.

Our Meta-analysis, which included the majority of participants, found that while the studies reported a lower incidence of IHPOD after TAVR, other studies reported a higher incidence. We analyzed potential links between modifiable delirium factors, the incidence of IHPOD after TAVR, and mean age, logistic EuroSCORE, or percentage of participants treated using TF access and observed no significant relationships among these factors. This suggests that these factors may not play a significant role in determining the incidence of IHPOD after TAVR as consistent with the findings of Abawi’s Meta-analysis conducted in 2017^69^, and other factors may have a stronger association.

The incidence of IHPOD in patients undergoing non-TF TAVR may be explained by various factors, including differences in patient profiles, comorbid conditions, medication side effects, and the invasive nature of non-TF TAVR. Factors such as general anesthesia, postoperative pain and opioid use, prolonged hospitalization, and systemic inflammation may also contribute to the increased incidence of IHPOD in non-TF TAVR compared to TF TAVR. Certain periprocedural or postprocedural factors such as stroke, cardiac tamponade, atrial fibrillation, and infections may also increase the risk of delirium after TAVR. The underlying mechanisms of delirium after TAVR may involve neurotransmitter imbalances, inflammatory processes, and physiological stress.

With the inclusion of a nationwide cohort study conducted by Stachon *et al*.^52^ in 2018 with 11 560 participants to the best of our knowledge, our meta-analysis is the first largest after Abawi’s and reports a significantly increased pooled estimate rate of IHPOD after TF TAVR and non-TF TAVR and for CAM-defined IHPOD. In contrast to the former, the pooled estimate rate of IHPOD significantly increased relative to the results of the previous one, after TF TAVR and non-TF TAVR and for CAM-defined IHPOD. The study’s larger sample size improves the precision of the association analyzed. The strength of this article is that it updates and consolidates the findings of the previous meta-analysis with a larger sample size, providing more precise estimates of the incidence of IHPOD after TAVR. However, the persistent heterogeneity suggests the need for further research in this area.

### Clinical implications for health managers and policymakers

Delirium should be assessed regularly, as a standard practice, since it is common after TAVR. Several risk factors cannot be modified (old age, preoperative comorbidities/conditions) but significant measures can be taken to prevent postoperative delirium after TAVR.

The identified modifiable risk factors for postoperative delirium following TAVR have significant clinical implications. Healthcare providers should prioritize preoperative cognitive screening to identify patients with pre-existing cognitive impairment, who are at higher risk of developing delirium. This can help implement interventions to reduce the risk of delirium, such as using alternative pain management strategies and avoiding benzodiazepines and opioids. The type of anesthesia should also be carefully considered, with regional anesthesia being preferred over general anesthesia.

Maintaining an appropriate electrolyte balance during and after the procedure is crucial to reducing the risk of delirium. In addition, inflammation may play a role in the development of delirium, and strategies to reduce inflammation, such as the use of corticosteroids or anti-inflammatory medications, may also help to reduce the risk of delirium. Healthcare providers should work together to create a multidisciplinary approach to patient care, incorporating these strategies to reduce the incidence of postoperative delirium and improve patient outcomes after TAVR.

Furthermore, healthcare providers should consider the use of an embolic protection device during TAVR, as cerebral diffusion weighted imaging lesions have been associated with IHPOD. Regular neurocognitive assessments of individuals undergoing TAVR are also recommended to evaluate the safety and effectiveness of the procedure, including different delirium assessment tools^70^. Nonpharmacological measures, such as managing sleep, anxiety, and agitation, encouraging mobility and self-care, and ensuring that patients have glasses, hearing aids, and dentures, can also be taken to prevent delirium in vulnerable individuals undergoing TAVR^71^. To minimize the risk of infections, it is important to maintain good hygiene practices and ensure the patient is stable in all aspects. While we may not be able to anticipate the development of infectious complications in advance, we can decide whether to proceed with TAVR immediately or to first treat any existing infection.

### Study limitations and future directions

Our study has certain limitations that must be taken into consideration when interpreting the findings. Firstly, we were limited by our primary studies, which lacked patient-level data (directly collected during a clinical trial). They also provided heterogeneous populations with varying ages, LES, the approach of the procedure, assessment, and comorbidities. All these factors could have contributed to the high heterogeneity in the pooled analysis of our study. Primary studies also did not provide data for the use of general versus local anesthesia, follow-up of mortality, postoperative complications, and the use of cerebral embolic protection. Hence, the association between these factors could not be assessed. Consequently, future studies are required to assess the role of anesthesia and cerebral embolic protection in preventing delirium after TAVR. Residual heterogeneity remained high even considering these variables together, suggesting that other features might have influenced the observed interstudy heterogeneity.

Furthermore, our results may underestimate the true incidence of delirium after TAVR due to the low sensitivity of the available screening tools and the difficulty in diagnosing the hypoactive subtype of delirium. There is limited information available on the clinical usefulness of the CAM method for identifying IHPOD in routine settings. Our meta-analysis relied on data from three large prospective registries, which did not provide clear information on the delirium diagnostic criteria and timing of the delirium screening, limiting the precision of our findings^38,46,52^. Hence, further research is needed to analyze the specificity and sensitivity of different diagnostic systems for delirium, such as the Diagnostic and Statistical Manual of Mental Disorders (DSM-5), DSM-IV, CAM, and Delirium Rating Scale-Revised-98 (DRS-R98). Such studies would help to develop effective methodologies for determining the incidence of IHPOD after TAVR.

## Conclusion

In conclusion, IHPOD following TAVR, was observed in 10.5% of individuals and 15.6% using CAM. Its incidence was found to be three times higher after non-TF TAVR (25.3%) compared to TF TAVR (9.3%). Stroke showed an incidence of 3.7% after TAVR and was found to be significantly associated with the risk of developing delirium following TAVR. Further studies are needed to evaluate possible causes and risk factors responsible for delirium and to assess the role of anesthesia and cerebral embolic protection in preventing delirium after TAVR. Previous literature consists of mostly observational studies with variable age, a small sample size with significant risk of bias and concerns on quality of evidence. Future research should contain more clinical trials and patient follow-up to access variable factors and indicators of developing delirium, accessing the quality of lifestyle of patients following TAVR and mortality associated with different procedural approaches and the role of preoperative modifiable factors.

## Ethical approval

Not applicable.

## Consent

Not applicable.

## Sources of funding

The author(s) received no financial support for the research, authorship, and/or publication of this article.

## Author contribution

S.O.: project administration, supervision, conceptualization, methodology, screening, data extraction, re-screening, statistical analysis, tables, manuscript writing, design, review-editing, formatting, and references; A.A.: methodology, screening, data extraction, tables, manuscript writing, and references; A.S.: methodology, screening, data extraction, and manuscript writing; A.K.: data extraction, tables, and manuscript writing; S.K.: data extraction, tables, manuscript writing; M.A.: re-screening, prisma flow chart, manuscript writing; M.H.A.: data extraction, statistical analysis, manuscript writing; M.A.A.: data extraction, statistical analysis, and manuscript writing.

## Conflicts of interest disclosure

The authors declare that they have no conflicts of interest.

## Research registration unique identifying number (UIN)

1. Name of the registry: not applicable.
2. Unique identifying number or registration ID: not applicable.
3. Hyperlink to your specific registration (must be publicly accessible and will be checked): not applicable.

## Guarantor

All authors accept full responsibility for the work and/or the conduct of the study, had access to the data, and controlled the decision to publish.

## Provenance and peer review

Not commissioned, externally peer-reviewed.

## Data availability statement

Available upon reasonable request from the corresponding author.
